# Primary seminoma of prostate in a patient with Klinefelter syndrome: A case report

**DOI:** 10.1097/MD.0000000000029117

**Published:** 2022-05-06

**Authors:** Duncheng Shi, Changjian Chen, Huagang Huang, Jingyu Tian, Jianfang Zhou, Shihua Jin

**Affiliations:** a Department of Urology, Xifeng People's Hospital, Huayuan East Road, Xifeng County, Guizhou Province, PR China; b Department of Urology, Shougang Shuigang General Hospital, Chayelin Road, Zhongshan District, Liupanshui City, Guizhou Province, PR China; c Department of Urology, Capital Medical University Affiliated Beijing Shijitan Hospital, No. 10 Yangfangdian Road, Haidian District, Beijing, PR China.

**Keywords:** klinefelter syndrome, prostate, seminoma

## Abstract

**Rationale:**

Klinefelter syndrome (KS) is a sex differentiation syndrome that occurs in men and is characterized by the 47XXY genotype. An association between KS and cancer has also been reported. The occurrence of seminoma of the prostate in KS has not been reported in the literature to date. Primary seminoma should be included in the differential diagnosis of prostate neoplasms in patients with KS.

**Patient concerns:**

A 39-year-old man presenting with urinary retention was admitted to our hospital. Physical examination revealed sparse pubic hairs, atrophic testes, and an underdeveloped penis. Hormonal examination revealed significantly lowered serum testosterone levels and markedly higher follicle-stimulating hormone levels. A chromosomal examination was performed. Computed tomography and magnetic resonance imaging imaging showed a neoplasm in the left lobe of the prostate, and immunohistochemical examination of a transrectal needle biopsy of the prostate was performed.

**Diagnoses:**

Chromosomal examination was exhibited a 47 XXY genotype. Histopathology and of Immunohistochemistry of the transrectal needle biopsy specimen confirmed a seminoma. No other neoplasm was found on systemic examination; therefore, the patient was diagnosed with primary prostate seminoma and Klinefelter syndrome.

**Interventions:**

The patient refused any treatment except catheterization because of religious reason.

**Outcomes:**

The patient died 2 years later.

**Lessons:**

Primary seminoma should be included in the differential diagnosis of neoplasms of the prostate in patients with KS. Transrectal ultrasound-guided prostate needle biopsy is essential for the diagnosis of prostate neoplasms, and cisplatin-based chemotherapy remains the primary treatment for seminoma.

## 1 Introduction

Klinefelter syndrome (KS) is a sex differentiation syndrome that occurs in male patients and is characterized by the 47XXY genotype; it exhibits clinical features including gynecomastia, eunuchoidism, small atrophic testes accompanied by aggregation of Leydig cells, hyalinization of the seminiferous tubules, and aspermatogenesis. Patients also have high serum and urinary gonadotropins.^[1]^ Patients with KS also show elevated risks for several types of cancers, including breast cancer, leukemia, and malignant germ cell tumors. The occurrence of a primary seminoma in KS is very rare, and only 3 cases of extragonadal seminomas have been reported. To our knowledge, primary seminoma of the prostate in KS has not been reported to date. In the present article, we report the first such case and review relevant literature.

## 2 Case presentation

**Figure 1:**
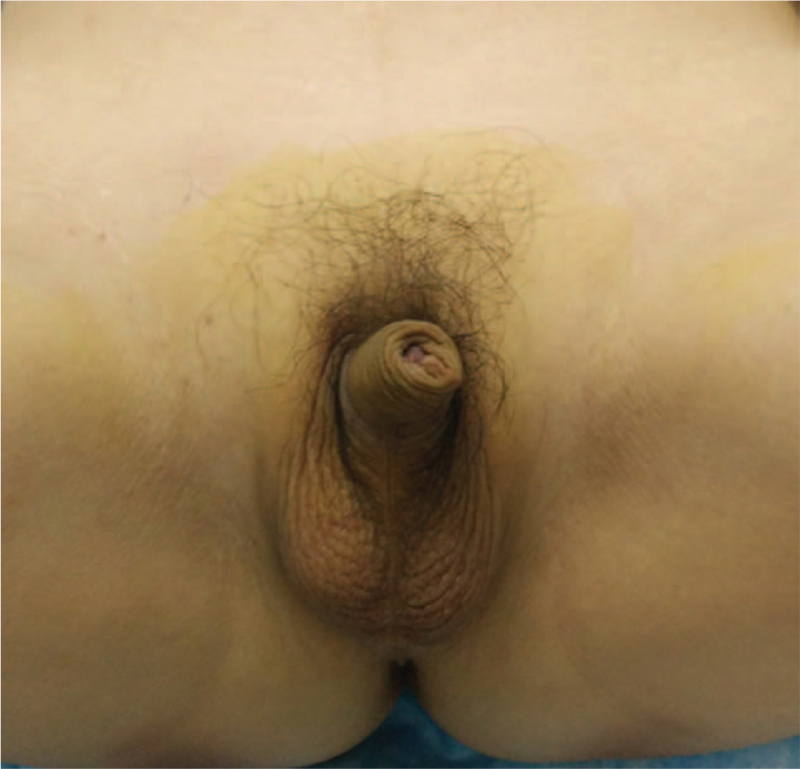
The patient had sparse pubic hair and undeveloped penis, and the bilateral testes were small and soft without palpable neoplasms.

**Figure 2:**
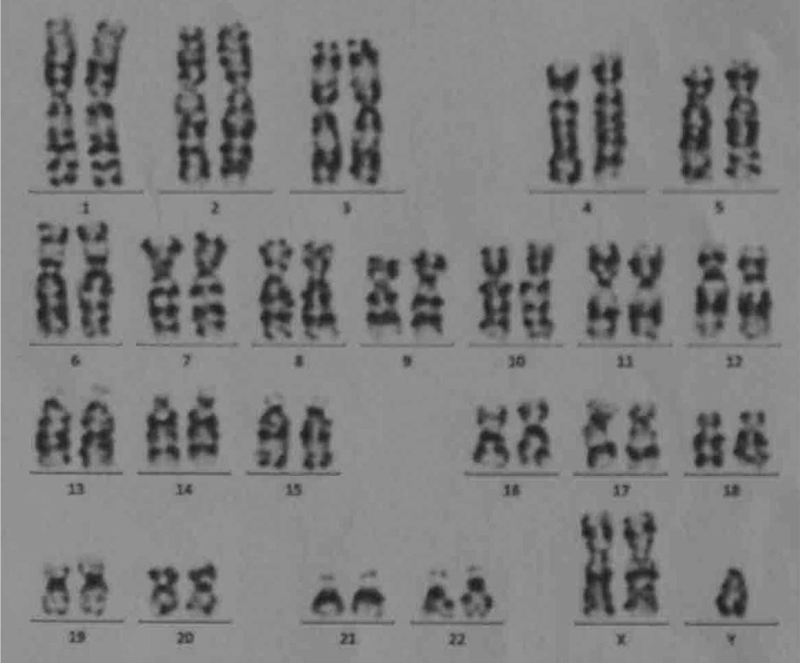
Chromosomal examination from peripheral blood cells revealed a 47XXY karyotype.

**Figure 3:**
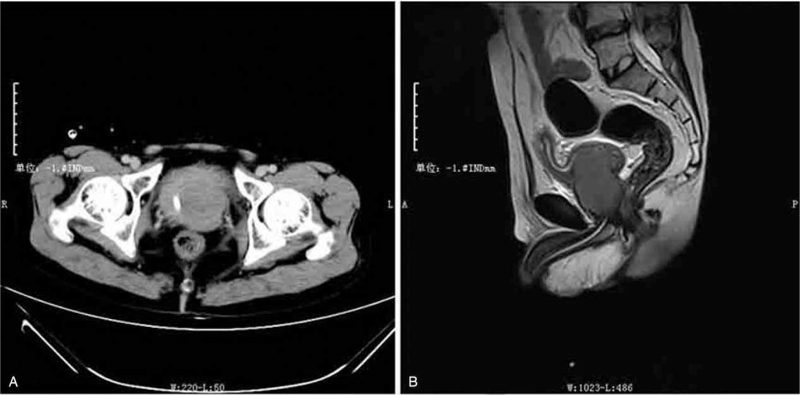
CT imaging (A) and T2 weighted MRI (B) revealed a 5.0 × 4.0 cm neoplasm of the left lobe of the prostate with invasion of the bladder neck and left seminal vesicle.

**Figure 4:**
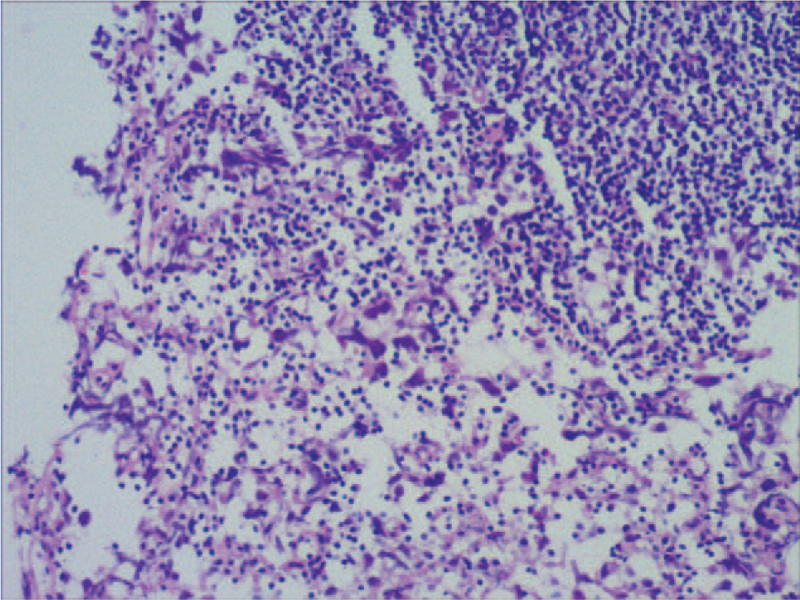
H&E staining of the biopsy samples exhibited tumor cells with atypia and large and hyperchromatic neculi (Figure 4A × 100; Figure 4B × 200).

**Figure 5:**
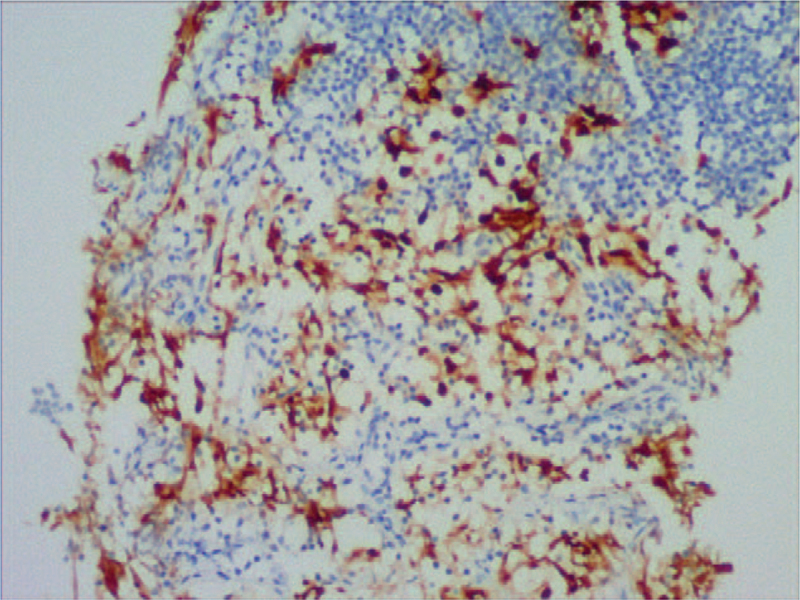
Immunohistologic image of OCT4 exhibited the positive staining of nuclei (Figure 5A × 100; Figure 5B × 200).

**Figure 6:**
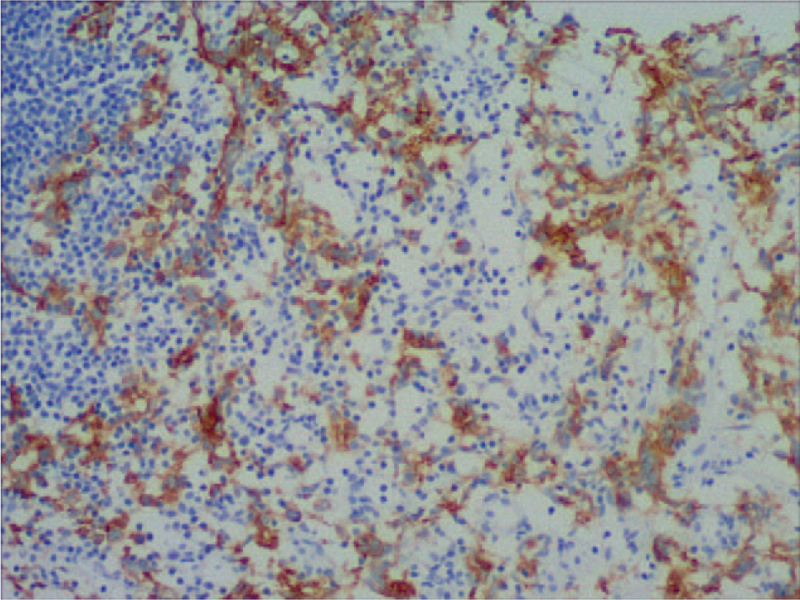
Immunohistologic image of CD117exhibited the positive staining of cytoplasm (Figure 6A × 100; Figure 6B × 200).

A 39-year-old man was admitted to our hospital with the chief complaint of urinary retention. Catheterization was performed on admission. The patient was 160.0 cm tall and weighed 61.5 kg. He had previously unrecognized *prominentia laryngea*, sparse pubic hair, an undeveloped penis, and atrophic testes without palpable neoplasms (Fig. 1). Digital rectal examination revealed a large, hard prostate gland without nodules. β-subunit human chorionic gonadotrophin and carcinoembryonic antigen concentrations were 0.6 mU/ml and 1.71 ng/ml, respectively--both in the normal range. The serum a-fetoprotein level was 4.58 IU/ml (normal range, 0–6.05) and prostate specific antigen (PSA) was 0.21 ng/ml (normal range, 0–4.00). Serum testosterone level was significantly decreased to 0.3 ng/ml (normal range, 2.2–10.5) while estradiol level was slightly elevated to 93.19 pg/ml (normal range, <87.00). Serum luteinizing hormone was 21.47 mIU/ml (normal range, 1.10–25.0), but the follicle-stimulating hormone level was markedly elevated to 38.9 mIU/ml (normal range, 1.1–11.8). Chromosomal examination of the peripheral blood cells revealed a 47XXY karyotype (Fig. 2). On the basis of these results, the patient was diagnosed with KS. A 5.0 × 3.5 cm low-echo-level neoplasm of the prostate was detected by ultrasonographic examination. Subsequent computed tomography (CT) imaging (Fig. 3A) and T2 weighted magnetic resonance imaging revealed a 5.0 × 4.0 cm neoplasm in the left lobe of the prostate with invasion of the bladder neck and left seminal vesicle (Fig. 3B). The neoplasm was unevenly enhanced on enhanced CT. No neoplasm was detected at other sites, including the mediastinum and retroperitoneal space, on chest and abdominal CT scans. Six core needle biopsies of the prostate were performed under transrectal ultrasonographic guidance. H&E staining of biopsy samples revealed tumor cells with atypia and large and hyperchromatic nuclei (Fig. 4). Immunohistochemical examination revealed a seminoma of the prostate, which stained immunochemically as follows: OCT4 (+) (Fig. 5), PLAP (+), SALL4 (+), CD117 (+) (Fig. 6), CD30 (−), serum alpha-fetoprotein (−), Ki67 (70%+), CAM5.2 (−), CD34 (−), SMA (−), Desmin (−), MyoD1 (−), S100 (−), GATA-3 (−), hepatocyte (−), and LCA (−).

Because no neoplasms were found in the testes by palpation or ultrasonography and no neoplasms were found in the mediastinal and retroperitoneal spaces on chest and abdominal CT scans, we diagnosed this case as a primary seminoma of the prostate. However, the patient refused any treatment except catheterization because of religious reasons. The patient died 2 years later because of tumor metastasis.

## 3 Discussion

KS was first described in 1942, and in 1959, genotype 47 XXY was discovered. Since our patient presented with some typical clinical features such as sparse pubic hair, atrophic testes, and an underdeveloped penis, we suspected gonadal insufficiency. Hormonal evaluation revealed significantly attenuated serum testosterone levels and significantly elevated follicle-stimulating hormone levels. Chromosomal examination revealed a 47XXY karyotype. We suggest that if the physical examination of a patient reveals the possibility of gonadal abnormality, hormonal and chromosomal assessments should be performed.

There is increased awareness of cancer susceptibility in individuals with KS. There has been a statistically significant increase in the mortality rates of lung cancer, breast cancer, and nonHodgkin lymphoma in patients with KS.^[2]^ The risk of testicular cancer in individuals with KS is of particular interest. Men with KS typically have atrophic testes and decreased androgen levels; however, no increased risk of testicular cancer has been found. Extraganodal germ cell tumors have also been associated with KS, with the majority located in the mediastinum.^[3]^ Williams et al reported that approximately one-third of men with mediastinal germ cell tumors had Klinefelter syndrome.^[4]^ The reason why germ cell tumors are associated with KS remains unclear. Sustained hypergonadotropism due to gonadal insufficiency may directly promote germ cell proliferation. This may explain the occurrence of malignant germ cell tumors in KS. Another possible explanation is the germ cell tumor susceptibility gene on the X chromosome.^[5]^ The genesis of extragonadal germ cell tumors is also hypothesized to be related to incomplete migration of primordial germ cells from the endoderm of the yolk sac to the gonads. Incomplete migration results in later malignant transformation to midline germ cell tumors along the urogenital ridge.^[3]^ Bonouvrie et al reviewed the literature from 1967 to March 2020; only 8 cases of extragonadal seminomas in KS patients have been reported; however, no seminoma of the prostate in KS was reported in the review.^[3]^

Because patients with KS have substantially decreased androgen levels, the risk of prostate cancer is significantly decreased.^[2]^ Primary extragonadal germ cell tumors of the prostate are very rare, with fewer than 20 cases described in the literature.^[6,7]^ Hashimoto et al reported a primary seminoma of the prostate in a 54 years old male, whose main complaint was difficulty with urination. They first suspected sarcoma of the prostate and were diagnosed with seminomas by needle biopsies of the prostate. Immunohistochemical examination revealed strong positivity for CD117 and periodic acid-Schiff staining.^[8]^ Primary nonseminoma germ cell tumors of the prostate in KS patients are rare, with only 2 cases reported (a yolk sac tumor and a yolk sac tumor + teratoma).^[9,10]^ To the best of our knowledge, the occurrence of seminoma of the prostate in KS has not been reported. Therefore, the present case is the first of its kind. This is also the third case of a germ cell tumor of the prostate in KS. The reason for the occurrence of primary seminoma in the prostate remains unclear. One possibility is that ectopic germ cells in the prostate that migrate from the yolk sac can develop into tumors. Another explanation is that pluripotent stem cells in the prostate transform neoplastic cells into germ cells.^[8]^

Seminoma of the prostate did not manifest any specific symptoms, and the main complaint in the present case was urinary retention. CT and magnetic resonance imaging scans of prostate neoplasms are often unevenly enhanced. Prostate sarcoma is the most common prostate tumor in young men. In these cases, the PSA level is usually normal, and digital rectal examination typically reveals a large, soft prostate. In the present case, we initially suspected sarcoma because of the patient's age and normal PSA level. However, the presence of a hard prostate, as revealed by digital rectal examination, did not support this diagnosis. The typical immunohistochemical markers of seminoma are OCT4 (+), PLAP (+), SALL4 (+), and CD117 (+). We performed a series of immunohistochemical tests on prostate needle biopsy samples and ultimately confirmed the diagnosis of seminoma. We did not detect any neoplasm in either testis by either palpation or ultrasonography after 6 months of follow-up (without additional treatment), and we therefore believe that this was a case of primary seminoma of the prostate instead of metastasis.

Cisplatin-based chemotherapy has been employed for extragonadal germ cell seminomas and has achieved excellent outcomes.^[10–13]^ Israel et al. treated 38 patients with extragonadal germ cell tumors with cisplatin-based chemotherapy, and 88.9% of patients with pure seminoma achieved a complete response and were disease-free after a median follow-up of 29 months.^[14]^ Stanton et al. also reported that 30 patients with advanced seminomas were treated with VAB-6. Complete remission was achieved in 24 of 28 evaluable patients. The median disease-free follow-up time for patients who achieved complete remission was 32 months. The authors concluded that VAB-6 is an effective treatment for patients with advanced seminomas. Similar chemotherapy was recommended as the initial therapy in all patients with stage II seminoma with disease larger than 5 cm, stage III seminoma, and extragonadal seminoma.^[15]^ However, the cytotoxicity of chemotherapeutic agents should be evaluated carefully. If the patient's response to chemotherapy is poor, surgery remains an important option. Radiation is also an effective treatment for testicular seminomas. However, the indications for adjuvant radiotherapy in the early stages of testicular germ cells are controversial. In contrast to the National Comprehensive Cancer Network (NCCN), the European Association of Urology (EAU) does not recommend radiotherapy for testicular seminoma, especially in men aged <40 years.^[16]^ However, intensity-modulated radiation therapy may still be a selective choice for primary mediastinal seminoma and massive pericardial effusion.^[17]^

Since the prognoses for extragonadal seminoma are associated with the clinical stage and site of the tumor, if the seminoma is combined with other tumors, the outcome may remain relatively poor.

## 4 Conclusions

We report the first case of primary seminoma of the prostate in an individual with KS. However, the reason for the rare incidence of seminomas associated with KS remains unclear. The possibility of seminoma should be considered when low urinary tract symptoms are the chief complaint of young men with KS. Transrectal ultrasound-guided prostate needle biopsy is essential for diagnosis and cisplatin-based chemotherapy remains the primary treatment.

## Author contributions

**Investigation:** Duncheng Shi, Changjian Chen, Huagang Huang.

**Methodology:** Duncheng Shi, Huagang Huang.

**Resources:** Jingyu Tian, Jianfang Zhou.

**Supervision:** Jingyu Tian, Shihua Jin.

**Writing – original draft:** Duncheng Shi, Changjian Chen, Shihua Jin.

**Writing – review & editing:** Shihua Jin.

## References

[1] Bonomi M Rochira V Pasquali D Balercia G Jannini EA Ferlin A. Klinefelter ItaliaN Group (KING). Klinefelter syndrome (KS): genetics, clinical phenotype and hypogonadism. J Endocrinol Invest 2017;40:123–34. 27644703 10.1007/s40618-016-0541-6 PMC5269463

[2] Swerdlow AJ Schoemaker MJ Higgins CD Wright AF Jacobs PA. UK Clinical Cytogenetics Group. Cancer incidence and mortality in men with Klinefelter syndrome: a cohort study. J Natl Cancer Inst 2005;97:1204–10. 16106025 10.1093/jnci/dji240

[3] Bonouvrie K van der Werff Ten Bosch J van den Akker M. Klinefelter syndrome and germ cell tumors: review of the literature. Int J Pediatr Endocrinol 2020;2020:18. 33005196 10.1186/s13633-020-00088-0 PMC7526209

[4] Williams LA Pankratz N Lane J. Klinefelter syndrome in males with germ cell tumors: a report from the Children's oncology group. Cancer 2018;124:3900–8. 30291793 10.1002/cncr.31667 PMC6241518

[5] Schneider DT Schuster AE Fritsch MK. Genetic analysis of mediastinal nonseminomatous germ cell tumors in children and adolescents. Gene Chromosome Canc 2002;34:115–25. 10.1002/gcc.10053 11921289

[6] Liu SG Lei B Li XN. Mixed extragonadal germ cell tumor arising from the prostate: a rare combination. Asian J Androl 2014;16:645–6. 24589456 10.4103/1008-682X.122871 PMC4104104

[7] Zheng W Wang L Yang D. Primary extragonadal germ cell tumor: a case report on prostate seminoma. Oncol Lett 2015;10:2323–6. 26622843 10.3892/ol.2015.3592 PMC4579977

[8] Hashimoto T Ohori M Sakamoto N Matsubayashi J Izumi M Tachibana M. Primary seminoma of the prostate. Int J Urol 2009;16:967–70. 20002841 10.1111/j.1442-2042.2009.02403.x

[9] Tay HP Bidair M Shabaik A Gilbaugh JH Schmidt JD. Primary yolk sac tumor of the prostate in a patient with Klinefelter's syndrome. J Urol 1995;153 (3 Pt 2):1066–9. 7853565

[10] Namiki K Tsuchiya A Noda K. Extragonadal germ cell tumor of the prostate associated with Klinefelter's syndrome. Int J Urol 1999;6:158–61. 10226829 10.1046/j.1442-2042.1999.06314.x

[11] Meares EM Briggs EM. Occult seminoma of the testis masquerading as primary extragonadal germinal neoplasms. Cancer 1972;30:300–6. 5040746 10.1002/1097-0142(197207)30:1<300::aid-cncr2820300142>3.0.co;2-x

[12] Daugaard G von der Maase H Olsen J Rørth M Skakkebaek NE. Carcinoma-in-situ testis in patients with assumed extragonadal germ-cell tumours. Lancet 1987;2:528–30. 2887831 10.1016/s0140-6736(87)92922-9

[13] Bastian PJ Skowasch D Bauriedel G. Primary extragonadal germ cell tumor of the prostate in a young man. Int J Urol 2004;11:671–3. 15285762 10.1111/j.1442-2042.2004.00861.x

[14] Israel A Bosl GJ Golbey RB Whitmore W Artini N. The results of chemotherapy for extragonadal germ-cell tumors in the cisplatin era: the Memorial Sloan-Kettering Cancer Center experience (1975 to 1982). J Clin Oncol 1985;3:1073–8. 2410574 10.1200/JCO.1985.3.8.1073

[15] Stanton GF Bosl GJ Whitmore WF. VAB-6 as initial treatment of patients with advanced seminoma. J Clin Oncol 1985;3:336–9. 2579214 10.1200/JCO.1985.3.3.336

[16] Jonska-Gmyrek J Peczkowski P Michalski W. Radiotherapy in testicular germ cell tumors - a literature review. Contemp Oncol (Pozn) 2017;21:203–8. 29180926 10.5114/wo.2017.69592 PMC5701577

[17] Cao J Zhou Y Zou F. Intensity modulated radiation therapy to treat primary female mediastinal seminoma and massive pericardial effusion: a case report. Oncol Lett 2017;13:1299–302. 28454250 10.3892/ol.2017.5555 PMC5403344

